# Nephroprotective Effect of Fennel (*Foeniculum vulgare*) Seeds and Their Sprouts on CCl_4_-Induced Nephrotoxicity and Oxidative Stress in Rats

**DOI:** 10.3390/antiox12020325

**Published:** 2023-01-30

**Authors:** Hassan Barakat, Ibrahim Ali Alkabeer, Sami A. Althwab, Hani A. Alfheeaid, Raghad M. Alhomaid, Mona S. Almujaydil, Raya S. A. Almuziree, Taqwa Bushnaq, Ahmed Mohamed

**Affiliations:** 1 Department of Food Science and Human Nutrition, College of Agriculture and Veterinary Medicine, Qassim University, Buraydah 51452, Saudi Arabia; 2 Food Technology Department, Faculty of Agriculture, Benha University, Moshtohor 13736, Egypt; 3 Department of Food Science and Nutrition, College of Science, Taif University, Taif 21944, Saudi Arabia; 4 Department of Biochemistry, Faculty of Agriculture, Benha University, Moshtohor 13736, Egypt

**Keywords:** *Foeniculum vulgare*, sprouts, bioactive components, oxidative stress, chronic disease, nephroprotection

## Abstract

Functional and nutritional characteristics of seed sprouts and their association with oxidative stress-related disorders have recently become a focus of scientific investigations. The biological activities of fennel seeds (FS) and fennel seed sprouts (FSS) were investigated in vitro and in vivo. The total phenolic content (TPC), total flavonoids (TF), total flavonols (TFF), and antioxidant activity (AOA) of FS and FSS were examined. HPLC and GC–MS analyses for FS and FSS were carried out. Consequently, the nephroprotective and antioxidative stress potential of FS and FSS extracts at 300 and 600 mg kg^−1^ on CCl_4_-induced nephrotoxicity and oxidative stress in rats was investigated. In this context, kidney relative weight, blood glucose level (BGL), lipid profile, kidney function (T. protein, albumin, globulin, creatinine, urea, and blood urea nitrogen (BUN)), and oxidative stress biomarkers (GSH, CAT, MDA, and SOD) in the rat’s blood as well as the histopathological alteration in kidney tissues were examined. Results indicated that the sprouting process of FS significantly improved TPC, TF, TFL, and AOA in vitro. HPLC identified nineteen compounds of phenolic acids and their derivatives in FS. Thirteen phenolic compounds in FS and FSS were identified, the highest of which was vanillic acid. Six flavonoids were also identified with a predominance of kaempferol. GC–MS indicated that the *trans*-anethole (1-methoxy-4-[(E)-prop-1-enyl]benzene) component was predominant in FS and FSS, significantly increasing after sprouting. In in vivo examination, administering FS and FSS extracts ameliorated the BGL, triglycerides (TG), total cholesterol (CHO), and their derivative levels compared to CCl_4_-intoxicated rats. A notable improvement in FS and FSS with 600 mg kg^−1^ compared to 300 mg kg^−1^ was observed. A dose of 600 mg FSS kg^−1^ reduced the TG, CHO, and LDL-C and increased HDL-C levels by 32.04, 24.62, 63.00, and 67.17% compared to G2, respectively. The atherogenic index (AI) was significantly improved with 600 mg kg^−1^ of FSS extracts. FS and FSS improved kidney function, reduced malondialdehyde (MDA), and restored the activity of reduced glutathione (GSH), superoxide dismutase (SOD), and catalase (CAT). Both FS and FSS extracts attenuated the histopathological alteration in CCl_4_-treated rats. Interestingly, FSS extract presented better efficiency as a nephroprotection agent than FS extract. In conclusion, FSS can potentially restore oxidative stability and improve kidney function after acute CCl_4_ kidney injury better than FS. Therefore, FS and FSS extracts might be used for their promising nephroprotective potential and to help prevent diseases related to oxidative stress. Further research on their application in humans is highly recommended.

## 1. Introduction

Oxidative stress is an imbalance between reactive oxygen species (ROS) production and detoxification or repair activities. ROS produce hydroxyl, superoxide, and hydrogen peroxide, which damage bases indirectly. Free radicals and hazardous peroxides harm lipids, proteins, and DNA when the cellular redox state is disrupted. When oxidative stress is present, it can interfere with the function of reactive oxidative species that serve as biological messengers in redox signaling. Researchers believe that oxidative stress may have a role in the onset of human diseases, including cancer and Alzheimer’s [1], atherosclerosis [2], and depression [3]. Scientists search for plant-based bioactive compounds to minimize oxidative stress. One option is to improve the nutritional quality of fennel and maximize its bioactive phytochemicals.

Kidney disease (CKD) is the 9th most significant cause of death, affecting 15% of US adults, and 37 million individuals [4]. Oxidative stress is a key element in the development of CKD. Several risk factors for the development of CKD have been identified, including oxidative stress, ectopic lipid buildup, dyslipidemia, renal cell injury, and dysfunction [5,6,7]. Remarkably, overproduced reactive oxygen species (ROS) and an inadequate antioxidant system result in an imbalanced redox, which is related to numerous pathological states and noninfectious chronic diseases, especially metabolic diseases, such as type 2 diabetes and CKD [5,8,9]. Surprisingly, in 2015, diabetes mellitus was identified as the leading cause of CKD, followed by high blood pressure and glomerulonephritis [10]. Historically, CCl_4_ has served various purposes, including as a grain fumigant, fire extinguisher fluid, metal degreaser, and dry cleaner [11]. It generates active free radicals, which cause severe diseases in organs and blood [12]. Lipid peroxidation induced by free radicals is thought to play a role in developing various pathological conditions [13]. CCl_4_-induced nephrotoxicity causes lipid peroxidation and the buildup of defective proteins, leading to kidney damage [14]. In recent years, the traditional use of medicinal plants has expanded substantially, and studies have proven their therapeutic efficacy against a wide range of diseases [15,16,17]. Fennel, a member of the Apiaceae family (Umbelliferaceae), is widely used as a medicinal herb [18,19]. It demonstrates hepatoprotective, antioxidant, antithrombotic, anti-inflammatory, antibacterial, and antifungal qualities [20,21]. Several antioxidant compounds have been shown to aid peripheral nerve regeneration after injury [22]. According to GC–MS examination, 31 fractions containing 99.46% fennel seeds were identified. The majority were for α-pinene, fenchone, 1-methoxy-4-prop-2-enylbenzene, (Z)-anethole, 1,8-cineole, 1-methoxy-4-prop-2-enylbenzene, anisaldehyde, and carvacrol [23]. Alam et al. [24] reported that 57 phytoconstituents were isolated from a methanolic extract of *F. vulgare* using GC–MS. The most abundant compounds were 1-methoxy-4-[(E)-prop-1-enyl]benzene (31.49%), 2-pentanone (25.01%), fenchone (11.68%), and benzaldehyde-4-methoxy (8.01%). Certain compounds were found in high concentrations, while others were in lesser concentrations. *F. vulgare* seed extracts have a wide range of bioactivity and practical applications [21,22,23,24].

*F. vulgare* has been shown to reduce neuronal toxicity by stabilizing the expression levels of amyloid precursor protein isoforms and oxidative stress indicators [25]. Due to its anticholinesterase activity, memory impairments associated with Alzheimer’s and dementia may benefit from *F. vulgare* extract [26]. The stress-relieving effects can lead to enhanced memory. Some antioxidants may help alleviate stress and its associated health problems [27]. In addition to halting the development of inflammatory diseases, a methanolic extract of *F. vulgare* seeds also provides central analgesic effects. Since *F. vulgare* has anti-inflammatory, analgesic, and antioxidative properties, it could be a valuable tool in the fight against peripheral nerve injury and its impact on recovery [28]. Organic extracts of fennel seeds are antimicrobial and assist healthy human growth [29]. The essential oil extracted from fennel seeds is also rich in antioxidants and has potent antibacterial effects [19,30,31,32]. In addition to these applications, the essential oil from fennel seeds has been shown to have pharmacological effects, such as analgesic, antidiabetic, anti-inflammatory, laxative, antitumor, anti-inflammatory, antispasmodic, antithrombotic, digestive, acaricidal, diuretic, nervous-disorder-curing, and hepatoprotective properties [19,32]. Other investigations have shown that essential fennel oil contains several compounds, including a-pinene, fenchone, anethole, and estragol [19,30,31,32]. For example, the aromatic monoterpene fenchone [33] has the potential to be chemically modified to treat tuberculosis as medicine [34]. The aromatic monoterpene 1-methoxy-4-[(E)-prop-1-enyl]benzene pharmacological effects include anti-inflammatory, neuroprotective, antinociceptive, anticonvulsant, amnesic, and anticancer properties [35,36].

The effects of reactive oxygen species (ROS) on multiple kidney functions have been documented [37]. Besides cell migration and proliferation, ROS molecules regulate apoptosis, inflammation, and extracellular matrix deposition. In the end, the impact of ROS on various kidney components is controlled by the local concentration of ROS, which is characterized by production and antioxidant response [38]. One of the primary mechanisms involved in redox signaling is the hydrogen peroxide oxidation of cysteine residues in proteins [39]. Cysteine residues exist as a thiolate anion (Cys–S-) at physiological pH, which is more prone to oxidation than the protonated cysteine thiol (Cys-SH) [40]. Nanomolar quantities of H_2_O_2_ oxidize thiolate anion to a sulfenic form (Cys–SOH), changing protein activity. Disulfide reductases TRX and GRX convert sulfenic anions to thiolate anions, restoring protein activity [41]. Higher H_2_O_2_ concentrations oxidize thiolate anions to sulfinic (SO_2_H) or sulfonic (SO_3_H) species. Sulfinic and sulfonic changes can be irreversible and cause lasting protein degradation; hence cells have antioxidant mechanisms. PRX and GPx enzymes limit intracellular H_2_O_2_ accumulation [37].

The bio-changes in phytochemicals during FS sprouting have not been studied until now. While FS extract has been shown to have nephroprotective effects in a few studies, more research is needed [42,43]. Research has not yet been conducted on FSS and their bioactivity on CCl_4_-induced nephrotoxicity and oxidative stress in rats. Therefore, analyses of total phenolic compounds (TPC), antioxidant activity (AOA), total flavonoids (TF), and total flavonols (TFL) were performed while the sprouts were developing. In addition, HPLC analyses of phenolics and GC–MS analyses of volatiles were conducted to learn more about bioactive compounds produced by the sprouting process. The antioxidative stress potential of selected FS and FSS extracts on CCl_4_-induced nephrotoxicity and oxidative stress in an animal model was investigated.

## 2. Materials and Methods

### 2.1. Raw Fennel Seeds

Fennel seeds (*Foeniculum vulgare* L.) were sourced from the Al-Tamimi market (https://www.tamimimarkets.com, accessed on 10 February 2021) in the Al Qassim region of Saudi Arabia. Qassim University, KSA’s College of Agriculture and Veterinary Medicine sent a plant expert (Dr. Mokded Rabhi) to verify the seeds’ legitimacy. Green fennel sprouts were produced by cleaning and sprouting healthy seeds to remove dust, damage, and sickness. The raw fennel seeds and freeze-dried sprouts were ground in an American-made laboratory mill (model ES2097) and then kept at 4 ± 1 °C until analysis.

### 2.2. F. vulgare Sprouting and Preparation of Aqueous and Ethanolic Extracts

Seeds were soaked in 1% sodium hypochlorite solution for 3 min before being sprouted in batches of 500 g. After being rinsed three times in sanitized distilled water (sd.H2O), the seeds were spread out evenly on 7 × 35 cm plastic trays and placed in a seed germinator. The seeds were germinated in a temperature-controlled seed germinator (Easygreen, Canada) with an atomizer at 17 ± 1 °C, 90–93% relative humidity, and faith light. For the first nine days, the fennel seeds were sprayed with 20.0 mL of sd.H_2_O/tray (day after day). Samples were collected every 3 days from the 1st day of germination to the 15th day. The fennel sprouts were frozen at −18 ± 1 °C for 24 h before being freeze-dried for 96 h at −48 °C and 0.032 mbar (CHRIST, Alpha 1–2 LD plus, Germany). Until the phytochemicals, HPLC, and GC–MS analyses, freeze-dried sprouts were pulverized in a small mill (Thomas-Wiley Mill, California, USA) and sieved (60 mesh sieve) to form a homogenous powder that was stored in dark containers at 4 ± 1 °C. Separately, 1 kg of FS was germinated under identical circumstances for 9 days, dried using the 24 h drying program described by Al-Qabba et al. [16], and then milled, sieved, and stored under cooled storage conditions until extraction. Ethanolic extracts (EE) were made by repeatedly extracting about one kilogram of FS or FSS in 5 L of ethanol (70%). In contrast, aqueous extracts (AE) were made by repeatedly extracting the same weight of FS or FSS in 5 L of hot water (70 °C for 10 min) three times. Both extracts were filtered, and the excess solvent or water was evaporated using a rotary evaporator set at 40 °C. After being frozen overnight, the residues were freeze-dried at −52 °C for 96 h under 0.032 mbar (CHRIST, Alpha 1-2 LD plus, Germany) [44]. The freeze-dried samples were pulverized with a porcelain cracker to make a uniform powder, which was then stored in dark packaging at 4 ± 1 °C until used.

### 2.3. Total Phenolic Content (TPC), Antioxidant Activity (AOA), Total Flavonoids (TF), and Total Flavonols (TFL) in Raw FS and during Sprouting

According to Yawadio Nsimba et al. [45], the TPC of FS and FSS using the Folin-Ciocalteu reagent protocol was determined, and TPC content was represented as mg of GAE g^−1^ DW. The radical scavenging activity (DPPH-RSA) of FS and FSS was spectrophotometrically determined according to Barakat and Rohn [46], and the results were expressed as micromoles of Trolox equivalents (TE) per gram (µmol TE g^−1^). The TF and TFL contents in the methanolic extract of FS and FSS were determined according to Mohdaly et al. [47] and Kumaran and Karunakaran [48], respectively. The data were presented as mg quercetin equivalent (QE) per g^−1^ (mg QE g^−1^).

### 2.4. Phenolics Quantification in FS and FSS Using HPLC-DAD

According to Kim et al. [49], phenolic compounds in FS and FSS were identified using an HPLC system HP1100 (Agilent Technologies, Palo Alto, CA, USA) equipped with an autosampler, quaternary pump, and diode array detector (DAD, Hewlett Packard 1050). The system used an Altima C18, 5 × 150 mm, 4.6 mm ID column, and an Altima C18, 5 mm guard column (Alltech). In the solvent system, the amounts of acetic acid were A (acetic acid 2.5%), B (acetic acid 8%), and C (Acetonitrile). In the extraction, (1:10) freeze-dried sample:70% methanol was applied, then the clear extract was evaporated under the N stream and re-solved in methanol, filtered, and injected. Ten microliters of the 70% methanolic extracted samples were injected at a flow rate of one milliliter per minute, and separation was completed at 25 °C. To quantify the phenolic compound, external standards’ retention time and mass spectra were compared to obtained peaks, and the results were presented as µg g^−1^.

### 2.5. Quantification of Volatile Components by GC–MS

Appropriate freeze-dried samples were extracted by 70% ethanol (GC grade), then the clear extract was evaporated under N stream, reconstituted in ethanol (GC grade), filtered, and then injected. The GC–MS analysis was performed using a Thermo Scientific Trace GC Ultra/ISQ Single Quadrupole MS, TG-5MS fused silica capillary column (30 m, 0.251 mm, and 0.1 mm film thickness) with a detection system that used an electron ionization device with an ionization energy of 70 eV. A steady stream of 1 mL min^−1^ of helium gas was used. The injector and MS transfer line temperatures were both maintained at 280 °C. The oven was programmed to heat from 50 °C (held for two minutes) to 150 °C at a rate of 7 °C min^−1^, then to 270 °C at a rate of 5 °C min^−1^ (held for two minutes), and lastly to 310 °C at a rate of 3.5 °C min^−1^ (held for 10 min). Relative peak area was used to quantify the observed components. Following the procedures laid out by Odeh and Allaf [23], retention times and mass spectra of obtained peaks and related data from the GC–MS analysis were compared with NIST and WILLY libraries to make a preliminary identification.

### 2.6. Animals and Experimental Design

Wistar rats (48 adult males) weighing 180–200 g were used. The Institutional Animal Ethics Committee (IAEC) of QU, KSA, approved this study (number 21-09-01 on Thursday, February 10, 2022). The laboratory conditions of 24 ± 1 °C and 40–45% relative humidity were maintained in polypropylene cages for the animals. Rats were acclimated for a week before being split into six groups of eight. The body weight of the rats was recorded and tagged for identifying purposes. The rats were given standard pellets and water *ad libitum* [50]. G1 (normal rats, NR) received olive oil (1.0 mL kg^−1^ twice weekly through intraperitoneal injection) and saline buffer (2 mL day^−1^ via oral gavage) for 6 weeks; G2 (CCl_4_-rats) received 2 mL of saline buffer orally/daily and an i.p. injection of a fresh mixture of CCl_4_ and olive oil (at a dosage of 1.0 mL kg^−1^) twice weekly [51]; G3 rats were given CCl_4_ i.p. twice weekly and 300 mg kg^−1^ of FS-AE orally/daily; G4 rats were given CCl_4_ i.p. twice weekly and 600 mg kg^−1^ of FS-AE orally/daily. G5 rats were given CCl_4_ i.p. twice weekly and 300 mg kg^−1^ of FSS-EE orally/daily; and G6 rats were given CCl_4_ i.p. twice weekly and 600 mg kg^−1^ of FSS-EE orally/daily. At the end of the sixth week, the animals fasted for 12 h with free access to water, then anesthetized with a mixture of Alcohol: Chloroform: Ether (1:2:3), according to Leila et al. [52], and sacrificed. To calculate the weight change %, rats were weighed after 12 h of fasting and immediately before slaughter. Blood samples were collected, and serum was separated for various biochemical examinations. A blood chemistry analyzer (HumaLyzer 4000, Germany) and necessary test kits were used to determine the biochemical parameters. The liver and kidneys were removed and weighed to calculate the relative weight of the organs.

#### 2.6.1. Determination of Kidney Function

Indicators of kidney function, such as total protein (T. protein, g dL^−1^), albumin (g dL^−1^), creatinine (mg dL^−1^), and urea (mg dL^−1^) concentrations were determined, respectively, using photometric, colorimetric test kits applying the Biuret method, photometric, colorimetric test kits applying the BCG method, photometric, colorimetric test kits, and fully enzymatic test kits applying the GLDH method, according to the instructions of the manufacturer. Globulin (g dL^−1^) was calculated by subtracting albumin from T. protein concentrations. Blood urea nitrogen (BUN, mg dL^−1^) was calculated by multiplying urea concentration by 0.47. All biochemical examination kits were purchased from Human Co., Wiesbaden, Germany. The atherogenic index (AI) was calculated according to Nwagha et al. [53].

#### 2.6.2. Oxidative Stress Biomarkers

Reduced glutathione (GSH) concentrations were determined using a GSH colorimetric assay kit (E-BC-K030-S, Elabscience, Texas, USA) in accordance with the protocol established by Beutler et al. [54]. Using a malondialdehyde (MDA, nmol mL^−1^) colorimetric test kit (E-BC-K025-S, Elabscience, Texas, USA), we quantified lipid peroxidation in terms of MDA content, as described by Ohkawa et al. [55]. The activity of superoxide dismutase (SOD, U L^−1^) was measured using a SOD typed activity assay kit (E-BC-K022-S, Elabscience, Texas, USA) in accordance with Giannopolitis and Ries [56]; results were expressed as U L^−1^. The activity of catalase (CAT, U L^−1^) was measured using a CAT activity assay kit (E-BC-K031-S, Elabscience, Texas, USA) according to the method of Aebi [57]. To determine the oxidative stress biomarkers in the kidney tissues, the appropriate weight of kidney tissue was homogenized in PBS (0.01 M, pH 7.4) on ice (1:9, w:v), then tissue homogenates were centrifuged (Sigma, Nussloch, Germany) under cooling at 10,000× *g* for 10 min. The clear supernatant was collected and preserved, and the GSH, MDA, SOD, and CAT concentrations were determined using the same kits. The blood chemistry analyzer (HumaLyzer 4000, HUMAN Gesellschaft für Biochemica und Diagnostica mbH, Max-Planck-Ring 21, 65205 Wiesbaden, Germany) measured all oxidative stress markers.

#### 2.6.3. Histopathological Studies

During autopsies, the kidneys of the rats were removed and preserved in 10% formalin saline for 24 h. After rinsing with municipal water, we dehydrated the specimens using a series of alcohol dilutions (methyl, ethyl, and absolute ethyl). After being cleaned in xylene, the specimens were baked in a hot air oven at 56 °C for 24 h to obtain embedding in paraffin. A sled microtome was used to cut 4-micron sections from tissue blocks. To facilitate a routine examination under an electric light microscope, tissue sections were routinely deparaffinized, mounted on glass slides, and stained with hematoxylin and eosin [58]. Four rats were randomly selected from each treated group, and the histological alterations were diagnosed using two experts with blind evaluation; their report was representatively shown.

### 2.7. Statistical Analysis

The statistical analysis was carried out using a one-way analysis of variance (ANOVA) using SPSS, ver. 22 (IBM Corp. New York, NY, USA, Released 2013). Data were treated as a complete randomization design, according to Steel et al. [59]. Multiple comparisons were carried out by applying the Duncan test, and the significance level was set at <0.05.

## 3. Results

### 3.1. Bioactive Compounds and Antioxidant Activity of FS and FSS

Table 1 shows the results of a quantitative study of phytochemicals in FS and FSS, including TPC, TF, TFL, and AOA measured by DPPH radical scavenging. According to Table 1, the total phenolic content (TPC) in FS was 70.42 mg GAE g^−1^. TF and TFL concentrations in FS were 4.83 and 4.92 mg QE g^−1^, respectively. In addition, DPPH-RSA was employed to track the development of antioxidant capacity. The research revealed that there was 9.36 mol of TE g^−1^ in FS. On day 3, there was already a noticeable drop in TPC, TF, TFL, and DPPH-RSA. With the progression of the sprouting period, the TPC, TF, TFL, and DPPH-RSA contents increased. The highest contents were observed on the 9th day. However, the TPC, TF, TFL, and DPPH-RSA contents were reduced on the 12^th^ and 15^th^ days.

### 3.2. Quantification of Phenolic Compounds in FS and FSS by HPLC-DAD

The quantitative analysis of phenolics in extracts of FS and FSS is presented in Table 2. Table 2 demonstrates the significant concentration of flavonoids in FS. Thirteen phenolic acids and six flavonoids were identified and quantified from FS and FSS. Most prevalent was vanillic acid (587.40 µg g^−1^); next came o-coumaric acid (112.77 µg g^−1^), and last came rosmarinic acid (64.41 µg g^−1^). Kaempferol (5913.55 µg g^−1^), resveratrol (472.19 µg g^−1^), rutin (423.28 µg g^−1^), myricetin (236.93 µg g^−1^), catechin (123.46 µg g^−1^), and quercetin (28.71 µg g^−1^) were the most common flavonoids discovered. On day 9 of sprouting, vanillic acid was the most abundant phenolic acid, but only 22% was retained. There was a 92.52% drop in o-Coumaric acid levels. Intriguingly, catechol, *p*-hydroxy benzoic acid, caffeic acid, chlorogenic acid, cinnamic acid, ellagic acid, ferulic acid, *p*-coumaric acid, benzoic acid, rosmarinic acid, and syringic acid contents were increased 7.80, 1.01, 1.80, 10.49, 2.01, 2.06, 2.42, 1.08, 3.63, 1.94, and 6.8-fold, respectively. In the same context, catechin, quercetin, and rutin were increased, whereas all flavonoids decreased on the 9th day of sprouting.

### 3.3. Determination of Volatile Component Concentrations in FS and FSS Using GC–MS

The volatile components of FS and FSS and their concentrations (%) are listed and tabulated in Table 3. Fifty and fifty-one components were found in FS and FSS, respectively (whole data were not shown, only concentrations more than 1% were presented). Eleven constituents with a concentration greater than 1% were found in the GC–MS analysis of FS. However, 1-methoxy-4-[(E)-prop-1-enyl]benzene (38.51%) was the most abundant, followed by estragol (23.65%), fenchone (11.18%), and 1-methylidene-4-prop-1-en-2-ylcyclohexane (7.17%). Newly synthesized components and shifts in the relative abundance of existing components were discovered through the GC–MS analysis of FSS. Interestingly, α-Pinene, 4-methoxybenzaldehyde, 1-methoxy-4-[(E)-prop-1-enyl]benzene, 2-hydroxy-2-(4-methoxyphenyl)acetic acid, and *cis*-13-Octadecenoic acid contents were increased. On the contrary, 1-methylidene-4-prop-1-en-2-ylcyclohexane, fenchone, 1-methoxy-4-prop-2-enylbenzene, 2-Methoxyphenyl)methyl acetate, 1-(3-hydroxy-4-methoxyphenyl)ethane-1,2-diol, and 10-Nonadecanone were decreased. The 4,5-dimethoxy-6-prop-2-enyl-1,3-benzodioxole and 7-Octadecenoic acid methyl ester were newly identified and quantified.

### 3.4. The Weight Gain, the Relative Weight of Organs, and Hypoglycemic Efficiency

Table 4 shows the results of an investigation into the effects of FS and FSS extracts on CCl_4_-induced nephrotoxicity and oxidative stress in rats, including their impact on weight gain, relative organ weight, and hypoglycemic efficiency. The injection of CCl_4_ had an immediate effect on the weight of the rats during the first month, and a subsequent slow weight gain % was observed in the 6th week in G2. Compared to G1, the administration of 600 mg kg^−1^ of FS or FSS at the end of the sixth week was the most effective treatment in restoring the rats’ weight. The weight gain was dose-related-enhanced and associatively reduced with either FS or FSS. The treated group showed statistically significant increases in organ weight relative to the control group. Positive attenuation was observed in rats treated with FS or FSS extracts. Table 4 demonstrates that after 6 weeks, both FS and FSS at 300 or 600 mg kg^−1^ significantly decreased FBG.

### 3.5. The Hypolipidemic Efficiency

Table 5 displays the findings of determining the hypolipidemic efficacy of FS and FSS at 300 and 600 mg kg^−1^ on CCl_4_-induced nephrotoxicity and oxidative stress in rats. CCl_4_-induced nephrotoxicity and oxidative stress considerably increased TG, CHO, LDL-C, and VLDL-C levels in rats. However, CCl_4_ injection resulted in a marked decline in HDL level compared to normal rats (G1). The lipid profile of dose-dependent manure was enhanced after administration of FS and FSS at 300 or 600 mg kg^−1^. A dose of 600 mg kg^−1^ of FS or FSS extracts was the most effective treatment for improving the blood profile, but this was not statistically significant compared to normal rats (G1). However, administrating FS and FSS at 300 or 600 mg kg^−1^ reduced the TG level by 10.97, 22.17, 26.57, and 32.04%, respectively. Interestingly, the rate of CHO reduction was 10.99, 32.43, 16.22, and 24.62% for treating rats with 300 and 600 mg kg^−1^ of FS and FSS extracts, respectively. After receiving FS and FSS at 300 and 600 mg kg^−1^, HDL-C increased by 40.27, 58.47, 14.31, and 67.17%, respectively, whereas LDL-C decreased by 45.58, 72.27, 26.03, and 63.00%, respectively. With therapy, the VLDL-C level rose in a dose- and treatment-dependent manner. There was a notable improvement in FS and FSS with 600 mg kg^−1^ compared to 300 mg kg^−1^. Injection of CCl_4_ resulted in a significant rise in AI in G2 compared to G1. It was found that FSS had more considerable mitigation of the atherogenicity problem than FS, especially when compared to G1. The best results were seen with 600 mg kg^−1^ of FSS extracts, which was not noticeably different from rats of G1.

### 3.6. The Kidney’s Functions

Table 6 shows the findings of an investigation into the nephroprotective efficacy of 300 and 600 mg kg^−1^ aqueous and ethanolic extracts of FS and FSS against CCl_4_-induced nephrotoxicity and oxidative stress in rats. Serum creatinine, urea, and BUN levels in G2 rats were significantly higher after CCl_4_ injection compared to NR in GI. In comparison, the levels of T. protein, albumin, and globulin were markedly decreased (Table 6). The dose-dependent elevations in creatinine, urea, and BUN that accompany CCl_4_ problems were significantly suppressed by either FS or FSS at 300 or 600 mg kg^−1^. Simultaneously, these doses brought the levels of T. protein, albumin, and globulin up to levels that were very close to those of G1 (Table 6). Compared to normal rats, the addition of FS or FSS at 600 mg kg-1 resulted in the most notable improvement.

### 3.7. Antioxidant Biomarkers

CCl_4_ injection significantly decreased GSH, CAT, and SOD levels and elevated MDA levels in the blood serum of G2 compared to normal rats in G1. Table 7 shows that the levels of MDA were significantly decreased, and the activity of the antioxidant enzymes GSH, CAT, and SOD were significantly increased in rats that were given FS or FSS at 300 or 600 mg kg^−1^. However, the autoxidation process was inhibited, and MDA levels were reduced after the administration of 300 mg kg^−1^ of FS or FSS, which was associated with a mild attenuation in GSH, CAT, and SOD. The most efficient treatment of FS or FSS was using a dose of 600 mg kg^−1^, which recorded an improvement rate of 40.08% and 37.87%, 37.17% and 46.52%, 114.56% and 154.13%, and 66.05% and 69.69% for GSH, DMA, CAT, and SOD when compared to the CCl_4_ group (G2), respectively.

Surprisingly, when comparing normal rats (G1) and CCl4-treated rats, the enzymatic defense system was significantly improved when rats were treated with 600 mg kg^−1^ of FSS extract, more significantly than with FS extract.

Table 8 demonstrates that when CCl_4_ was injected into G2 rats, the GSH, CAT, and SOD levels considerably decreased. In contrast, the MDA level was elevated compared to the levels in the tissue of normal rats (G1). Antioxidant activities presented in GSH, CAT, and SOD, as well as MDA levels, were significantly enhanced in rats treated with FS or FSS at 300 or 600 mg kg^−1^ (Table 8). However, the autoxidation process was inhibited, and MDA levels were reduced after the administration of 300 mg kg^−1^ FS or FSS, eliciting considerable attenuation in GSH, CAT, and SOD. The most effective dose for treating FS or FSS was 600 mg kg^−1^, with increases in GSH, CAT, and SOD of 41.05% and 37.85%, 115.63% and 153.13%, and 67.24 and 70.69%, respectively, compared to the CCl_4_-group (G2). The MDA level was reduced by 37.20% and 46.52% for FS and TSS treatments, respectively. The treatment with 600 mg kg^−1^ FSS extracts considerably outperformed the FS extract in boosting the enzymatic defense system in rats compared to normal rats (G1) and CCl_4_-treated rats (G2).

### 3.8. Renal Histoarchitecture

**Figure 1:**
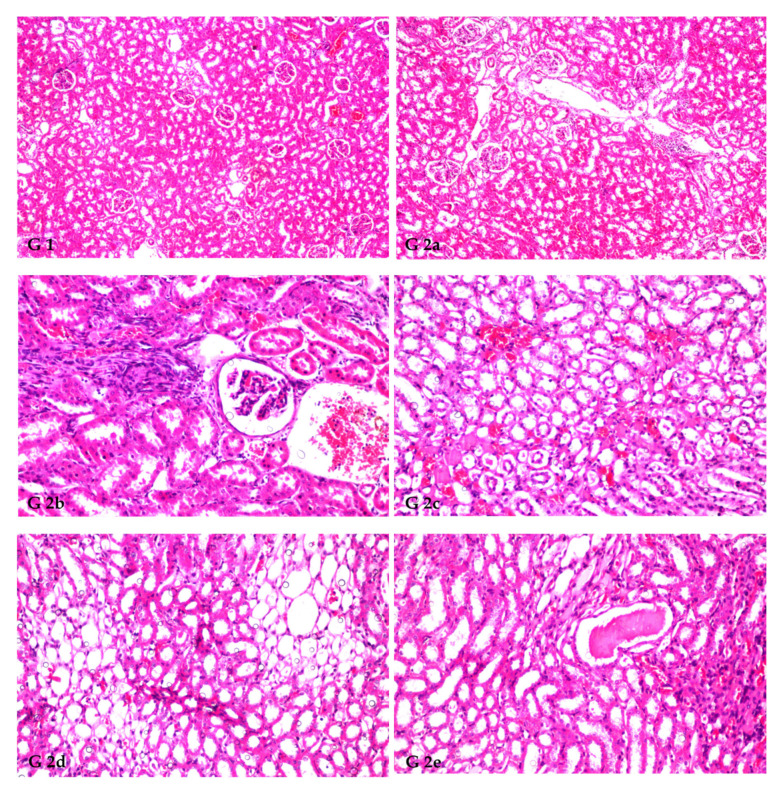

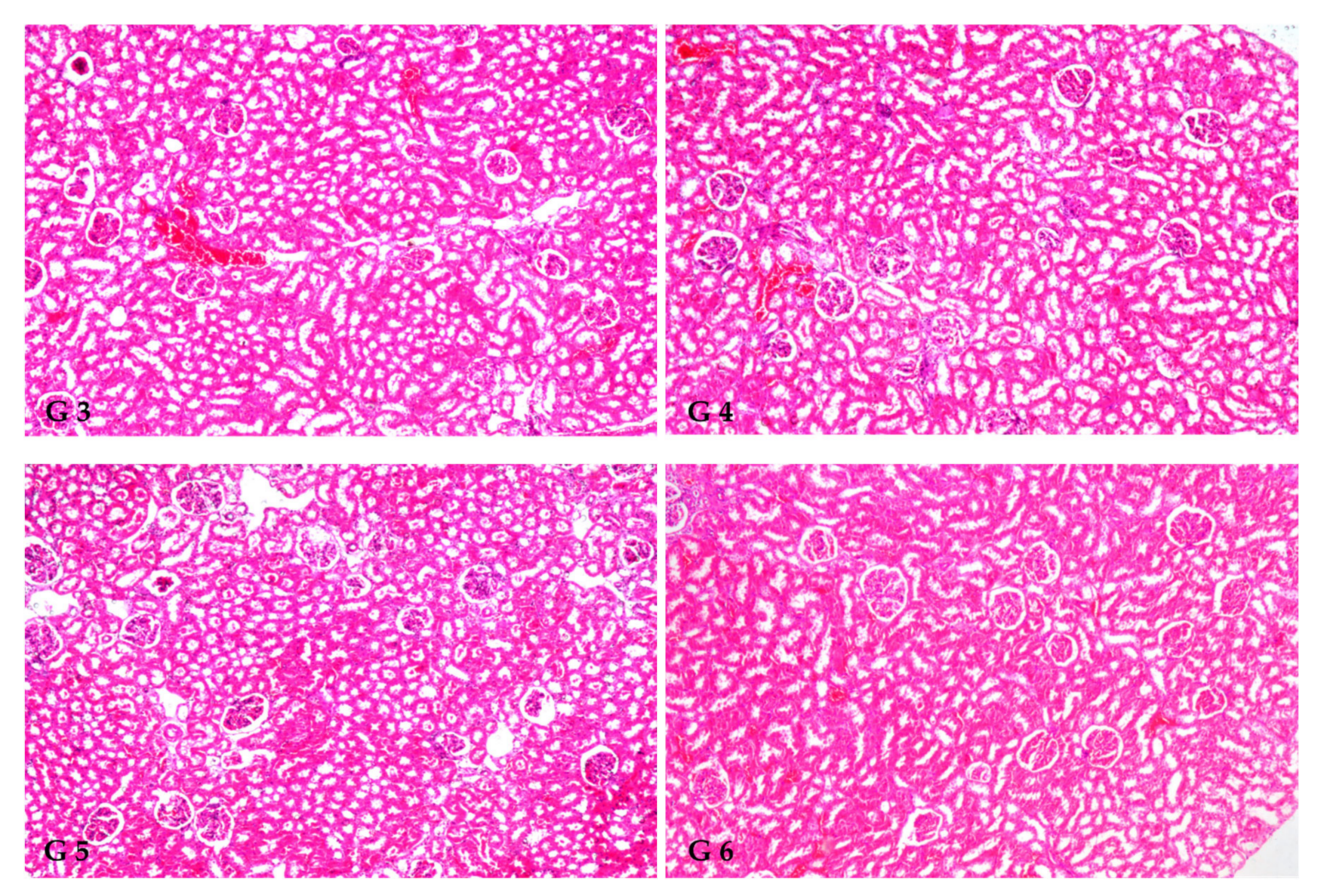
Histopathological findings in rat kidneys treated by FS and FSS extracts along with CCl_4_ nephrotoxicity induction (X16 and X40). GI: Show normal histological structure of the glomeruli and tubules at the cortex, as recorded (**G1**). The histoarchitecture of the CCl_4_-treated rats (G2) showed focal inflammatory cell infiltration at the cortex surrounding the glomeruli and blood vessels as well as between the tubules (**G2a**), focal fibrosis with atrophy and obliteration of the tubules with dilatation of the blood vessels at the cortex (**G2b**), corticomedullary portion showed focal hemorrhages between the obliterated tubules (**G2c**), focal cystic dilatation with flattened lining epithelium in others (**G2d**), and eosinophilic casts formation was detected in the lumen of the cystic tubules at the corticomedullary adjacent to the focal fibrosis (**G2e**). G3 (300 mg kg^−1^ aqueous extract of FS) shows congestion in the cortical blood vessels (**G3**). In (**G4**) (600 mg kg^−1^ FS of aqueous extract), (G5, 300 mg kg^−1^ of FSS alcoholic extract), and (**G6**, 600 mg kg^−1^ of FSS alcoholic extract) showed no histopathological alteration as recorded in (**G4**, **G5**, and **G6**), respectively.

The histopathology confirmed the biochemical findings, as currently remarked. Figure 1 demonstrates the histological alterations in rat kidneys of treated rats treated with FS and FSS extracts. In this study, the kidneys of the control group (GI) had normal glomeruli and tubules at the cortex (Figure 1, G1). The histoarchitecture of the CCl_4_-treated rats (G2) showed focal inflammatory cell infiltration at the cortex surrounding the glomeruli and blood vessels and between the tubules (Figure 1, G2a). There was also focal fibrosis with atrophy and obliteration of the tubules with dilatation of the blood vessels at the cortex (Figure 1, G2b). The corticomedullary portion showed focal hemorrhages between the obliterated tubules (Figure 1, G2c) and focal cystic dilatation with flattened lining epithelium in others (Figure 1, G2d). Eosinophilic cast formation was detected in the lumen of the cystic tubules at the corticomedullary adjacent to the focal fibrosis (Figure 1, G2e). In G3, when 300 mg kg^−1^ aqueous extract of FS was given, congestion was observed in the cortical blood vessels (Figure 1, G3). However, administrating 600 mg kg^−1^ FS aqueous extract (G4) and both 300 and 600 mg kg^−1^ FSS alcoholic extract, as marked in (G5 and G6), respectively, showed no histopathological alteration as recorded in (Figure 1, G4, G5, and G6).

## 4. Discussion

Antioxidant substances such as phenolic compounds have been praised for their ability to neutralize free radicals, including hydrogen peroxide, hydroxyl radicals, and superoxide anion, which have been linked to metabolic diseases [60,61]. The current study aimed to provide primary research on the bio-changing and destiny of active compounds in fennel sprouts, such as polyphenols and volatiles. This study reveals that fennel seed sprouts may offer a novel source of antioxidant-rich active compounds [62,63]. Interestingly during sprouting, phenolics and antioxidants increased [63,64]. Washing and soaking reduced TPC and AOA at the start of sprouting. Osmotic pressure may cause bioactive chemical leaching. This damaged the TPC and AOA [63,65]. Our data showed a 1.26-fold rise in TPC by day 6, indicating bioactive molecule production. The AOA rose 1.52 times from day 3 to day 6. With sprouting, TPC and AOA increased significantly, and also newly produced TPC-enhanced AOA, which has antioxidative and ameliorative properties [16,66,67].

Meanwhile, during the sprouting process, new phenolics, flavonoids, and flavonols were produced [68,69,70]. The number and content of phenolic compounds in seeds can be modified by genotype (species/variety), soil, environmental conditions, harvest ripeness, postharvest storage conditions, and extraction procedures [71]. Recent research confirms that consuming sprout extracts may assist in decreasing cellular oxidation [16,72]. A higher concentration of phenolics was found in *F. vulgare* sprouts compared to seeds as the sprouting time rose, findings which are consistent with those of Swieca and Gawlik-Dziki [64] with superior flavonoid content [73].

HPLC examination revealed the presence of 13 different phenolic compounds, with vanillic acid ranking as the most abundant. Six flavonoids were found, with kaempferol ranking highest, which differed from Odeh and Allaf [23] and Roby et al. [74]. Raw fennel seed-phenolic levels vary, and sprouting affects flavonoids and phenolic acids. Fennel seed and sprout phenolic content was measured at 0, 6, and 9 days. Vanillic acid was the major acid, although, in other investigations, it was rare [70,75,76]. Caffeic acid, p-coumaric acid, and rosmarinic acid declined in 6-day sprouts and then increased by 80%, 7%, and 93%, respectively. Ferulic acid more than doubled in 9-day sprouts from 20.01 to 48.51 µg g^−1^. The results were higher than those obtained by Odeh and Allaf [23], who found that ferulic acid content in fennel seeds was 2.31 μg g^−1^. Increases in phenolics may be related to sprouting enhancing phenolic acid degradation and extraction and phenolic compound production [77]. No research has confirmed bio-changes in sprouting fennel seeds. However, increasing and reducing phenolic acid content changed the phenolic profile [16,71]. Kaempferol, resveratrol, quercetin, catechin, myricetin, and rutin were detected and ranked, with kaempferol being the highest and quercetin the lowest, giving results more elevated than those obtained by Mohamad et al. [78] and Allaithy [79].

Eleven components with concentrations greater than 1% were detected in the GC–MS analysis of FS. However, the predominant component was 1-methoxy-4-[(E)-prop-1-enyl]benzene (38.41%). For the most part, our findings coincided with those of other researchers; however, the precise amounts varied slightly [19,32]. This was also closely indicated by the results of Suleiman and Helal [80], who identified 1-methoxy-4-[(E)-prop-1-enyl]benzene (31.49%) as predominant. Consequently, sprouting for 9 days led to elevated levels of 4-Methoxybenzaldehyde, 1-methoxy-4-[(E)-prop-1-enyl]benzene, and 2-hydroxy-2-(4-methoxyphenyl)acetic acid in FSS. This investigation found and quantified 4,5-dimethoxy-6-prop-2-enyl-1,3-benzodioxole and 7-octadecenoic acid methyl ester. Hong et al. [81] also indicated that 1-methoxy-4-[(E)-prop-1-enyl]benzene and fenchone were the most abundant fennel essential oil volatiles. No research has been carried out on FS phytoconstituents and volatiles. Thus, future research can be guided by this work. Research is necessary to determine the physiological reactions induced by sprouting, significantly when volatile substances change the chemical formula, rearrangement, and elongation of the carbon chain or derivatives.

For CCl_4_-induced nephrotoxicity and oxidative stress in rats, both FS and FSS at 300 and 600 mg kg^−1^ showed hypolipidemic efficacy. The lipid profile was shown to be enhanced in dose-dependent manure. To improve lipid profiles, 600 mg kg^−1^ of FS and FSS extracts were the most effective treatment with no significant difference compared to normal rats. Interestingly, CCl_4_ injection (G2) resulted in substantial increases in AI compared to normal rats. Rats treated with FSS at 600 mg kg^−1^ showed more attenuation of atherogenicity than those treated with FS compared to normal rats. Afiat et al. [82] remarked a modest improvement in LDL-C, triglyceride, and HDL values after consuming fennel.

Similarly, Zakernezhad et al. [83] showed that fennel extract affected leptin receptor expression, leading to a more favorable lipid profile. The authors recommended that fennel can treat hyperlipidemia instead of using chemical drugs with adverse effects. Conversely, essential fennel oil prevented lipid and metabolic abnormalities in high-fat diet-induced obese rats through inhalation—potentially beneficial effects in metabolic health and reversal of diet-induced obesity [81].

The administration of FS and FSS had considerably attenuated kidney function when the impact of the researched therapies on renal functions was examined. Markers of the kidneys and histological alterations in renal tissues were found to show similar results [42,43]. Al-Amoudi explained that the normal histological structure of the kidney cortex was restored, and normal values of creatinine and urea nitrogen were restored after the co-treatment of albino rats with sodium valproic and fennel oil [84]. The albumin or T.Protein/creatinine ratios were attenuated when rats were treated with 300 and 600 mg kg^−1^ of FS or FSS extracts. A possible explanation for CCl_4′_s toxic impact on kidney function is the oxidative stress caused by the compound’s high production of reactive oxygen species. As a result, it was hypothesized that the antioxidant activity of FS and FSS extract contributed to its protective effect against CCl_4_ toxicity in the kidney [42,43]. Irazabal and Torres [37] confirmed that organic compounds, such as ascorbic acid, α-tocopherol, carotenoids, flavonoids, and reduced GSH, work efficiently as a nonenzymatic antioxidant system to compact ROS. Excess albumin in the urine is a sign of kidney disease known as Albuminuria [37,85]. Albuminuria is associated with increased renal endothelial permeability and endothelial dysfunction [86]. Lower albumin and creatinine contents in the blood indicated their higher content in urine because of occurred leakage [37]. It could be noticed that albumin or T.Protein/creatinine ratios were attenuated when rats were treated with 300 and 600 mg kg^−1^ of FS or FSS extracts. Exogenous antioxidants from herbal extracts reduce dialysis-related oxidative stress and inflammation [87]. Much clinical research suggests that herbal antioxidants improve dialysis outcomes [43,88,89]. Such data can shed light on herbal antioxidant processes and medical consequences. Resveratrol neutralizes free radicals, reducing oxidative stress [90]. Quercetin contains antioxidant, antidiabetic, analgesic, antihistaminic, antiviral, cholesterol-lowering, and renal hemodynamic modulating effects [91]. The potential processes and medical implications of herbal antioxidants seem more convoluted; additional research is needed [87].

Marked by the catabolite malondialdehyde, lipid peroxidation increases the risk of tissue damage due to the released ROS [92]. Glutathione (GSH) is an antioxidant that is present in all mammalian cells, despite not being an enzyme. When oxidized to GSSG, GSH acts as a cofactor for numerous detoxification enzymes (GPx, GST, and others), protecting cells from oxidative stress and keeping cellular redox balanced [93]. Again, SOD acts as a catalyst, converting two molecules of the harmful superoxide anion (*O_2_) into harmless hydrogen peroxide (H_2_O_2_) and oxygen (O_2_). Thus, the detrimental effects of superoxide anion are mitigated [94]. MDA, a primary by-product of lipid peroxidation, is a crucial indication of oxidative stress. ROS increases tissue damage risk and causes lipid peroxidation, as measured by the catabolite malondialdehyde [92]. Previous studies, including chronic i.p. injection of CCl_4_, found that SOD, CAT, GPx, and GSH activities significantly decreased while the MDA level was significantly raised [16,95].

Flavonoids, a class of phenolic found in abundance in FS and FSS, have antioxidative properties and are thought to provide functional and therapeutic effects, as demonstrated in this study’s first portion [28,96]. The most common phenolics in this analysis were vanillic acid and kaempferol, both of which have the potential as protective agents [97,98,99]. GC–MS testing also revealed that 1-methoxy-4-[(E)-prop-1-enyl]benzene and fenchone, accounted for 38.41 and 11.18%, respectively. SOD, CAT, and glutathione peroxidase (GPx), as antioxidant enzymes, play an important role in ridding the body of free radicals [100]. In blood, the most efficient treatment of FS or FSS was by using a dose of 600 mg kg^−1^, which recorded an improvement rate of 40.08 and 37.87%, 37.17 and 46.52%, 114.56 and 154.13%, and 66.05 and 69.69% for GSH, DMA, CAT, and SOD when compared to the CCl_4_ group (G2), respectively. While comparing to the CCl_4_ group (G2), FS or FSS used in a dose of 600 mg kg^−1^ increased levels of GSH, DMA, CAT, and SOD in kidney tissues by 41.06 and 37.85%, 37.20 and 46.53%, 115.20 and 153.13%, and 67.24 and 70.69%, respectively. Fascinatingly, when comparing normal rats (G1) and CCl_4_-treated rats, the enzymatic defense system was considerably improved when rats were treated with 600 mg kg^−1^ of FSS extract rather than with FS extract. According to Rather et al. [20], numerous therapeutic applications for *F. vulgare* have been validated by in vitro and in vivo models. Recently, Samadi-Noshahr et al. [101] demonstrated that FS and 1-methoxy-4-[(E)-prop-1-enyl]benzene could protect the liver from diabetes-induced hepatic damage in rats, probably via hypoglycemic and antioxidant effects. The plant-based extracts show antioxidative and nephroprotective activities in rats with renal injury [42,43]. Their bioactive compounds ameliorate oxidative stress-induced kidney damage, enhance the antioxidant system, and decrease the inflammatory process and fibrosis, most likely by activating the KEAP1/Nrf2/ARE pathway and by deactivating the NFB pathway [102].The activation of Nrf_2_ results in the upregulation of several antioxidant enzymes and cytoprotective genes, such as SOD, CAT, glutathione peroxidase (GPx), and heme oxygenase-1(HO-1). Several Nrf_2_ activators have been proven effective at activating Nrf_2_ signaling through different mechanisms in both in vitro and in vivo models of diabetes [103]. It was found that the activation of the Nrf_2_–AREpathway led to the quenching of ROS overproduction caused by advanced glycation end products (AGEs) [104]. Nrf_2_ is the key to regulating GSH levels by upregulating enzymes involved in GSH synthesis. Nrf_2_ increases glutamate cysteine ligase catalysis (GCL), the rate-limiting step in GSH synthesis [105]. Under physiological conditions, Nrf_2_ locates in the cytoplasm and combines with its inhibitor Kelch-like ECH-associated protein-1 (KEAP1). During oxidative stress, Nrf_2_ is free from KEAP1 and binds to the genes encoding antioxidant enzymes in the nucleus to increase their expression [106,107]. It was found that Nrf_2_ has a protective effect on high glucose-induced oxidative damage in cultured cells and diabetic complications in animal models [108]. Hyperglycemia activates the Nrf2 pathway through the generation of ROS in the kidney tissue of humans or animals with diabetic nephropathy and tissue cultures [109,110,111]. Peixoto et al. [112] concluded that an imbalance in renal redox status is associated with markers of renal injury in the early stage of diabetes millets and hypertension. However, antioxidant treatment reestablished the redox status and prevented oxidative stress-induced renal damage.

Histopathological kidney findings are congruent with biochemical estimates. CCl4 injection (G2) induced inflammation around glomeruli, blood arteries, and tubules. Dogukan et al. [113] observed similar histopathological alterations in the renal tissue of rats in response to CCl_4_. Histological alterations may also be produced by functional overloading of nephrons, leading to renal failure [86] and/or free radical production via CCl_4_ metabolism [114,115]. FS and FSS extracts help restore the kidney from the destructive effects of CCl_4_. This may be due to FS and FSS (as potent antioxidants) acting on reactive oxygen species (ROS) induced by CCl_4_ [116]. The phenolic components in *F. vulgare* extracts have an antioxidative role and free radical scavenging properties, which allows them to reduce CCl_4_-induced acute nephrotoxicity [52]. Our results are consistent with other researchers who found that plant extracts with varying molecular compositions have pharmacological effects by restoring normalcy after CCl_4_ abuses were introduced [52,117].

## 5. Conclusions

This study revealed that FS and FSS have antioxidative properties. Extracts from both FS and FSS are rich in phenolic and volatile components, mainly flavonoids with significant antioxidant activity. Flavonoids were detected in significant proportions in *F. vulgare* sprouts, according to a phenolic analysis, providing validity to stated functional and therapeutic effects of the plant. In HPLC analysis, thirteen phenolic compounds, with a predominance of vanillic acid, and six flavonoids, with a predominance of kaempferol, were discovered. GC–MS analysis revealed that the predominant component was 1-methoxy-4-[(E)-prop-1-enyl]benzene (38.41%), followed by *trans*-anethole (benzene, 1-methoxy-4-(2-propenyl)) (23.65%), fenchone (11.18%), and 1,7-octadiene, 2-methyl-6-methylene-cyclohexene (7.17%). The administration of FS and FSS extracts improved BGL, TG, CHO, and their derivatives in the in vivo studies compared to CCl_4_-intoxicated rats. Furthermore, they suppressed renal function, lowered MDA, and revived GSH, SOD, and CAT. In rats exposed to CCl4, histological alterations were reduced by FS and FSS extracts. Contrary to predictions, FSS extract revealed higher activity as a nephroprotection agent than FS extract. Finally, FSS may restore oxidative stability and enhance kidney function after acute CCl_4_ renal damage compared to FS. Further work on using FS and FSS extracts in humans is recommended owing to their potential nephroprotective qualities and capacity to help prevent diseases related to oxidative stress.

## Institutional Review Board Statement

The study was approved by the committee of Research Ethics, Deanship of Scientific Research, Qassim University (21-09-01 on Thursday, February 10, 2022), SA. The approval was governed by the Control and Supervision of Experiments on Animals (CPCSEA) Committee of the National Committee of Bioethics (NCBE), which implements regulations related to the ethics of research on living creatures.

## Figures and Tables

**Table 1:** Total phenolic content, total flavonoids, total flavonols, and potential antioxidant capacities of FS during sprouting at 17 ± 1 °C and 90–93% RH (mean ± SE), *n* = 6.

Item	Sprouting Period (Day)					
	Raw Seeds	3	6	9	12	15
TPC (mg GAE g^−1^)	70.42 ±3.28 ^a^	40.56 ±2.70 ^d^	51.10 ±3.79 ^c^	75.20 ±4.48 ^a^	62.04 ±2.12 ^b^	54.48 ±3.70 ^c^
TF (mg QE g^−1^)	4.83 ±0.45 ^a^	2.82 ±0.14 ^d^	4.29 ±0.21 ^b^	5.18 ±0.21 ^a^	3.86 ±0.25 ^c^	3.23 ±0.24 ^c^
TFL (mg QE g^−1^)	4.92 ±0.23 ^a^	3.22 ±0.23 ^c^	3.62 ±0.29 ^c^	4.54 ±0.38 ^ab^	4.39 ±0.29 ^b^	3.02 ±0.31 ^c^
DPPH (µmol of TE g^−1^)	9.36 ±0.07 ^b^	6.51 ±1.09 ^d^	9.91 ±1.29 ^b^	11.93 ±1.92 ^a^	8.02 ±0.06 ^c^	8.67 ±0.22 ^c^

TPC: total phenolic content; TF: total flavonoids; TFL: total flavonols; DPPH: antioxidant activity using DPPH assay; RH: relative humidity; ^a,b,c,d^: the values with the same superscripted letters in the same row are not significantly different at *p* < 0.05.

**Table 2:** Quantitative analysis of phenolic compounds in *F. vulgare* during sprouting at 17 ± 1 °C and 90–93% RH (mean ± SE), *n* = 3.

Item	No.	Compound	Phenolics (µg g^−1^) *	
			Sprouting Period (Day)	
			Raw Fennel Seed	9-Day Sprouts
Phenolic acids	1	Pyrogallol	-	-
	2	Quinol	-	-
	3	3-Hydroxytyrosol	-	-
	4	Catechol	9.26 ± 0.23 ^b^	72.26 ± 5.41 ^a^
	5	p-Hydroxy benzoic acid	32.25 ± 2.57 ^a^	32.60 ± 3.58 ^a^
	6	Caffeic acid	26.72 ± 5.98 ^b^	48.22 ± 7.25 ^a^
	7	Chlorogenic acid	6.79 ± 3.25 ^b^	71.25 ± 9.25 ^a^
	8	Cinnamic acid	9.38 ± 0.98 ^b^	18.83 ± 5.14 ^a^
	9	Ellagic acid	25.35 ± 4.98 ^b^	52.30 ± 8.24 ^a^
	10	Vanillic acid	587.40 ± 31.09 ^a^	129.08 ± 5.97 ^b^
	11	Ferulic acid	20.01 ± 2.49 ^b^	48.51 ± 4.75 ^a^
	12	Gallic acid	-	-
	13	*O*-coumaric acid	112.77 ± 5.68 ^a^	8.44 ± 1.28 ^b^
	14	*p*-coumaric acid	18.46 ± 2.97 ^a^	19.89 ± 2.58 ^a^
	15	Benzoic acid	30.38 ± 3.97 ^b^	110.35 ± 12.32 ^a^
	16	Rosmarinic acid	64.41 ± 6.17 ^b^	124.71 ± 15.28 ^a^
	17	Syringic acid	9.72 ± 0.98 ^b^	66.08 ± 10.28 ^a^
Flavonoids	1	Catechin	123.46 ± 19.27 ^b^	151.46 ± 20.27 ^a^
	2	Kaempferol	5913.55 ± 152.93 ^a^	2357.57 ± 251.71 ^b^
	3	Myricetin	236.93 ± 12.59 ^b^	166.94 ± 23.19 ^a^
	4	Quercetin	28.71 ± 1.25 ^b^	192.35 ± 18.97 ^a^
	5	Rutin	423.28 ± 20.02 ^b^	985.29 ± 12.35 ^a^
	6	Resveratrol	472.19 ± 25.98	402.24 ± 32.19 ^b^
	7	Naringenin	-	-

*: Phenolic acids were identified at 280 nm and flavonoids were identified at 365 nm, -: Not detected, ^a,b^: the values with the same superscripted letters in the same row are not significantly different at *p* < 0.05.

**Table 3:** GC–MS analysis of volatile compounds in *F. vulgare* seeds and their 9-day sprouts at 17 ± 1 °C and 90–93% RH, *n* = 2.

RT	MW	Compound	Concentration %	
			Raw Fennel Seed	9-Day Sprouts
6.51	152	α-Pinene	0.44	1.08
9.19	136	1-methylidene-4-prop-1-en-2-ylcyclohexane	7.17	3.32
10.94	152	Fenchone	11.18	7.42
14.30	148	1-methoxy-4-prop-2-enylbenzene	23.65	22.89
15.89	136	4-methoxybenzaldehyde	1.45	2.32
15.87	144	Phenylhydrazine hydrochloride	1.81	-
16.80	148	1-methoxy-4-[(E)-prop-1-enyl]benzene	38.41	42.32
23.92	150	2-hydroxy-2-(4-methoxyphenyl)acetic acid	1.65	3.34
25.00	222	4,5-dimethoxy-6-prop-2-enyl-1,3-benzodioxole	-	4.23
26.10	180	2-Methoxyphenyl)methyl acetate	1.80	0.51
26.84	165	1-(3-hydroxy-4-methoxyphenyl)ethane-1,2-diol	1.86	0.52
34.20	296	7-Octadecenoic acid methyl ester	-	0.89
35.70	282	*cis*-13-Octadecenoic acid	3.12	6.42
48.78	152	10-Nonadecanone	1.57	0.53

**Table 4:** The effect of FS and FSS extracts on weight gain %, organ weight, and FBG in CCl_4_-induced nephrotoxicity and oxidative stress in rats (mean ± SE), *n* = 8.

Groups	Weight Gain %		Organ Relative Weight (%)		FBG
	Week 3	Week 6	Liver	Kidneys	
G1	38.11 ±2.66	52.69 ±2.13	3.24 ±0.22 ^a^	0.79 ±0.06 ^a^	77.90 ±2.81^a^
G2	16.42 ±5.26	24.45 ±3.48	3.71 ±0.17 ^b^	0.84 ±0.03 ^a^	91.24 ±6.87 ^b^
G3	25.33 ±5.37	35.45 ±6.19	3.24 ±0.04 ^a^	0.67 ±0.03 ^ab^	81.55 ±2.87 ^a^
G4	35.50 ±4.88	62.17 ±4.58	2.91 ±0.24 ^a^	0.66 ±0.05 ^b^	79.99 ±6.69 ^a^
G5	31.73 ±3.43	55.19 ±4.44	3.15 ±0.16 ^a^	0.78 ±0.02 ^a^	82.83 ±10.74 ^a^
G6	40.51 ±5.26	66.27 ±5.21	2.96 ± 0.09 ^a^	0.74 ±0.02 ^a^	78.83 ±3.44 ^a^

G1-G6: experimental groups, see Materials and Methods, Section 2.7; FBG: fasting blood glucose level measured in blood serum of 12 h fasted rats; ^a,b^: there is no significant difference (*p* > 0.05) between any two means within the same column that have the same superscripted letters.

**Table 5:** Effect of FS and FSS extracts on lipid profile and atherogenic index in CCl_4_-induced nephrotoxicity and oxidative stress in rats (mean ± SE), *n* = 8.

Groups	Lipid Profile Parameters					
	TG	CHO	HDL-C	LDL-C	VLDL-C	AI
G1	35.76 ±2.85 ^b^	69.40 ±5.20 ^b^	35.38 ±3.04 ^ab^	26.86 ±6.52 ^bcd^	7.15 ±0.57 ^c^	0.005 ±0.004 ^d^
G2	53.59 ±5.53 ^a^	89.29 ±4.23 ^a^	23.33 ±2.56 ^c^	55.24 ±3.83 ^a^	10.72 ±1.10 ^a^	0.361 ±0.024 ^a^
G3	47.71 ±4.30 ^ab^	72.33 ±4.53 ^b^	32.73 ±3.84 ^abc^	30.06 ±5.50 ^bc^	9.54 ±0.86 ^ab^	0.164 ±0.014 ^b^
G4	41.71 ±4.08 ^ab^	60.33 ±4.35 ^b^	36.67 ±4.41 ^ab^	15.32 ±3.96 ^d^	8.34 ±0.82 ^abc^	0.056 ±0.011 ^c^
G5	36.42 ±4.31 ^b^	74.81 ±6.15 ^b^	26.67 ±2.89 ^bc^	40.86 ±6.74 ^ab^	7.28 ±0.86 ^c^	0.135 ±0.012 ^b^
G6	39.35 ±3.30 ^b^	67.31 ±3.37 ^b^	39.00 ±5.15 ^a^	20.44 ±4.61 ^cd^	7.87 ±0.66 ^bc^	0.004 ±0.006 ^d^

TG: triglycerides; CHO: total cholesterols; HDL-C: high-density lipoprotein-cholesterols; LDL-C: low-density lipoprotein-cholesterols; VLDL-C: very low-density lipoprotein cholesterols; AI: atherogenic index; ^a,b,c,d^: there is no significant difference (*p* > 0.05) between any two means within the same column with the same superscripted letters.

**Table 6:** The effect of FS and FSS extracts on kidney function in CCl_4_-induced nephrotoxicity and oxidative stress in rats (mean ± SE), *n* = 8.

Group	Kidney Function					
	T. Protein (g dL^−1^)	Albumin (g dL^−1^)	Globulin (g dL^−1^)	Creatinine (mg dL^−1^)	Urea (mg dL^−1^)	BUN (mg dL^−1^)
G1	8.05 ± 0.25 ^a^	3.27 ± 0.11 ^a^	4.78 ± 0.21 ^a^	1.13 ± 0.16 ^c^	48.79 ± 3.09 ^b^	22.93 ± 1.45 ^b^
G2	6.26 ± 0.10 ^b^	2.60 ± 0.07 ^c^	3.67 ± 0.08 ^b^	2.00 ± 0.10 ^a^	60.42 ± 3.66 ^a^	28.40 ± 1.72 ^a^
G3	7.36 ± 0.46 ^a^	2.86 ± 0.19 ^bc^	4.50 ± 0.47 ^a^	1.57 ± 0.06 ^b^	51.93 ± 5.27 ^ab^	24.41 ± 2.48 ^a^
G4	7.85 ± 0.16 ^a^	3.33 ± 0.11 ^a^	4.52 ± 0.10 ^a^	1.26 ± 0.08 ^c^	44.36 ± 3.59 ^b^	20.85 ± 1.69 ^b^
G5	7.28 ± 0.20 ^b^	3.12 ± 0.10 ^ab^	4.16 ± 0.17 ^a^	1.27 ± 0.02 ^c^	46.28 ± 1.56 ^b^	21.75 ± 0.74 ^b^
G6	7.81 ± 0.68 ^a^	3.38 ± 0.13 ^a^	4.43 ± 0.75 ^a^	1.16 ± 0.07 ^c^	45.58 ± 1.44 ^b^	21.42 ± 0.68 ^b^

^a, b & c^: no significant difference (*p* > 0.05) between any two means within the same column with the same superscripted letters.

**Table 7:** The effects of FS and FSS extracts on antioxidant biomarkers in the blood serum of CCl_4_-induced nephrotoxicity and oxidative stress in rats (mean ± SE), *n* = 8.

Group	Antioxidant Biomarkers			
	GSH (µg dL^−1^)	MDA (nmol mL^−1^)	CAT (U L^−1^)	SOD (U L^−1^)
G1	47.60 ± 4.89 ^a^	7.20 ± 0.43 ^cd^	46.69 ± 5.59 ^b^	73.32 ± 1.07 ^b^
G2	36.63 ± 4.50 ^b^	12.51 ± 0.51 ^a^	23.83 ± 4.09 ^c^	46.75 ± 1.93 ^d^
G3	43.56 ± 2.03 ^ab^	9.35 ± 0.64 ^b^	45.61 ± 5.71 ^b^	64.00 ± 0.92 ^c^
G4	51.31 ± 2.66 ^a^	7.86 ± 0.36 ^cd^	51.13 ± 1.61 ^ab^	77.63 ± 1.58 ^a^
G5	43.73 ± 4.43 ^ab^	8.57 ± 0.67 ^bc^	44.09 ± 6.60 ^b^	67.44 ± 1.23 ^c^
G6	50.50 ± 1.58 ^a^	6.69 ± 0.35 ^d^	60.56 ± 4.26 ^a^	79.33 ± 1.58 ^a^

GSH: reduced glutathione; MDA: malonaldehyde; CAT: catalase; SOD: superoxide dismutase; ^a, b, c, and d^: no significant difference (*p* > 0.05) between any two means within the same column with the same superscripted letters.

**Table 8:** The effects of FS and FSS extracts on antioxidant biomarkers in kidney tissue of CCl_4_-induced nephrotoxicity and oxidative stress in rats (mean ± SE), *n* = 4.

Group	Antioxidant Biomarkers			
	GSH (µg g^−1^)	MDA (n mol g^−1^)	CAT (U g^−1^)	SOD (U L^−1^)
G1	6.90 ± 0.54 ^a^	8.28 ± 0.47 ^c^	0.63 ± 0.06 ^bc^	0.96 ± 0.08 ^a^
G2	5.31 ± 0.44 ^c^	14.38 ± 0.50 ^a^	0.32 ± 0.04 ^d^	0.58 ± 0.09 ^b^
G3	6.36 ± 0.18 ^b^	10.75 ± 0.57 ^b^	0.61 ± 0.05 ^c^	0.80 ± 0.08 ^b^
G4	7.49 ± 0.27 ^a^	9.03 ± 0.37 ^c^	0.69 ± 0.02 ^b^	0.97 ± 0.06 ^a^
G5	6.34 ± 0.35 ^b^	9.85 ± 0.53 ^bc^	0.59 ± 0.05 ^c^	0.84 ± 0.08 ^b^
G6	7.32 ± 0.12 ^a^	7.69 ± 0.40 ^d^	0.81 ± 0.04 ^a^	0.99 ± 0.12 ^a^

GSH: reduced glutathione; MDA: malonaldehyde; CAT: catalase; SOD: superoxide dismutase; ^a, b, c, and d^: no significant difference (*p* > 0.05) between any two means within the same column with the same superscripted letters.

## Data Availability

Data are contained within the article.

## Abbreviations

AOA, antioxidant activity; CAT, catalase; CHO, total cholesterol; DPPH: 1,1-diphenyl-2-picryl hydrazine; dw: dry weight; FBG, fasting blood glucose; FS, fennel seeds; FSS, fennel seeds sprouts; GA: gallic acid; GAE: gallic acid equivalent; GSH: reduced glutathione; HDL-C, high-density lipoproteins; LDL-C, low-density lipoproteins; MDA: malonaldehyde; QE: quercetin equivalent; RSA: radical scavenging activity; SE: standard error; SOD: superoxide dismutase; TBA, thiobarbituric acid; TC: total carotenoids; TE: Trolox equivalents; TF: total flavonoids; TFL, total flavonols; TG, triglycerides; TPC, total phenolic compounds; VLDL-C, very low-density lipoproteins.
